# Whole-genome sequence of *Macaca fascicularis*: liver tissue

**DOI:** 10.1186/s12863-023-01114-9

**Published:** 2023-02-14

**Authors:** Eun-Hye Seo, Jeong-Hwan Kim, Da-Hee Kim, Jung-Hwa Oh, Seon-Young Kim

**Affiliations:** 1 Korean Bioinformation Center, Korea Research Institute of Bioscience and Biotechnology, Daejeon, Republic of Korea; 2 Aging Convergence Research Center, Korea Research Institute of Bioscience and Biotechnology, Daejeon, Republic of Korea; 3 Jeonbuk Branch, Korea Institute of Toxicology, Jeonbuk, Republic of Korea; 4 Department of Predictive Toxicology, Korea Institute of Toxicology, Daejeon, Republic of Korea

**Keywords:** *Macaca fascicularis*, Cynomolgus monkeys, Whole genome sequencing

## Abstract

**Objectives:**

Thrombocytopenia is a condition that causes a low amount of blood platelets. Platelets are blood cells that play an essential role in blood coagulation. Therefore, thrombocytopenia can put the patient at risk for mild to severe bleeding. Thrombocytopenia is caused by a decrease in platelet production in the bone marrow or by a drug or immune system problem when production is normal. In particular, in some ASO-induced thrombocytopenia, the mechanism is not clear. Therefore, whole genome sequencing (WGS) was performed to discover genetic differences that affect thrombocytopenia and individual susceptibility to drugs between normal and reduced platelet monkeys despite administering the same ASO.

**Data description:**

Three antisense oligonucleotide (ASO) substances were injected into the subcutaneous tissue of monkeys for 12 weeks in two experiments. The monkeys were classified into three groups: monkeys with thrombocytopenia, monkeys without thrombocytopenia, and control monkeys not treated with ASO substances. Whole genome sequencing data was generated using liver tissues of monkeys. These data will be useful for identifying genetic differences that affect thrombocytopenia and drug sensitivity.

**Supplementary Information:**

The online version contains supplementary material available at 10.1186/s12863-023-01114-9.

## Objective

Platelets, also known as thrombocytes, are one type of blood cell involved in blood clotting and hemostasis. They are formed in the bone marrow, a spongelike tissue in the bone. Usually, platelet count is 150,000—400,000 per microliter of blood. Thrombocytopenia is diagnosed when platelet count is reduced to less than 150,000 per microliter [1]. The low amount of platelets in the blood increases the risk of bleeding, leading to life-threatening bleeding such as cerebral hemorrhage. Thrombocytopenia is caused by several mechanisms, including decreased platelet production, increased splenic sequestration, and increased platelet destruction [2]. Platelet production is reduced in the bone marrow as a representative cause among them. When production is normal, thrombocytopenia is caused by drug or immune system problems.

Antisense oligonucleotides (ASO) are single-stranded DNA-based disease-modifying drugs that bind specifically to complementary mRNA and regulate protein expression through several mechanisms such as mRNA degradation, splicing modulation, and steric block of the ribosome [3–5]. It is already used to treat various diseases [5–12]. However, recent toxicity studies have shown that ASO substances cause thrombocytopenia in proportion to drug concentration, and some drugs also cause severe platelet reduction at low concentrations. However, the mechanism for this problem is not yet clear. This data investigates the genetic differences in individual sensitivity to drugs and platelet reduction in the monkey liver. Specifically, these data compare genomic differences among monkeys with varying degrees of reduction in platelet counts despite the same ASO administration.

The data was initially produced to elucidate the genetic differences for drug toxicity, but they were not published due to the small number of samples and the limitations of observing significant genetic differences responsible for differential drug toxicity. However, since it contains a lot of information on the animal model used in this experiment, it will be helpful to researchers using the animal model.

## Data description

We used Cynomolgus monkeys (*Macaca fascicularis*) with a high level of genetic homology to humans for our experiments. We used three ASO substances to confirm the effect of ASO on thrombocytopenia. Cynomolgus monkeys were purchased from NafoVanny (Tam Phuoc Hamlet, Bien Hoa City, Dong Nai Province, Vietnam) and animal study carried out at Korea Institute of Toxicology (KIT, Daejeon, Korea), an accredited animal facility, complying with the AAALAC International Animal Care Policies. The Animal Care and Use Committee of the KIT reviewed and approved all the study protocols. Imported monkeys were SRV, SIV, STLV and B-virus free and quarantined at the nonhuman primate facility at KIT for at least 30 days. Before the ASO treatment, animals were permitted an acclimation period of 1 day to the laboratory environment. The ASO substance was administered to the subcutaneous tissue of monkeys for 12 weeks. The dose volume for each animal was calculated using the most recently measured body weight (i.e. weekly body weight measurements) at a volume of 0.4 mL/kg. It was administered once every two days in the first week and then once a week from the following weeks. We prepared five Cynomolgus monkeys and two ASO substances in the first experiment. One monkey was a control that was not treated with the drug, and the other four monkeys were treated with ASO #1 and #2 at a dosage of 25 mg/kg and 30 mg/kg, respectively. Blood samples were collected from animals via cephalic vein for evaluation of hematology including platelets counts. Then, the number of platelets was measured and classified into Normal and LowPLT (TCP) groups. (Table 1, Additional file 1) [13]. We prepared 12 Cynomolgus monkeys in the second experiment and treated them with ASO #3 dose-dependently. Doses ranged from 0 to 40, and the number of platelets was measured and classified into normal and LowPLT(TCP) groups (Table 1, Additional file 2) [14]. The animals were euthanized by exsanguination while under deep anesthesia prior to necropsy. Liver tissues were collected for NGS analysis and the samples were flash frozen in liquid nitrogen at necropsy.

**Table 1:** Overview of Additional files/data set

Label	Name of Additional file/data set	File Types (file extension)	Data repository and identifier (DOI or accession number)
Additional file 1	1st analysis PLT counts	Dataset (.xlxs)	FigShare (https://doi.org/10.6084/m9.figshare.19736242) [13]
Additional file 2	2nd analysis PLT counts	Dataset (.xlxs)	FigShare (https://doi.org/10.6084/m9.figshare.19738171) [14]
Additional file 3	Quality and quantity of the sequencing data	Dataset (.xlxs)	Figshare (https://doi.org/10.6084/m9.figshare.19738336) [15]
Data set	1st analysis and 2nd analysis	Fastq file (fastq.gz)	Korea Nucleotide Archive (Accession no.: KRA2200718) [16] Korea Nucleotide Archive (Accession no.: KRA2200719) [17] Sequence Read Archive (https://identifiers.org/ncbi/insdc.sra:SRP409432) [18]

Total genomic DNA was extracted from monkey liver tissues using the DNeasy Blood and Tissue Kit (Qiagen). The sequencing library was prepared using the Illumina TruSeq Nano DNA library prep kit (Illumina). Sequencing was performed based on the Hiseq X Ten platform (Illumina) to generate 150 bp paired-end reads. The sequenced reads were mapped to the rheMac3 reference genome using the BWA-MEM algorithm (v0.7.12-r1039) [19]. The resulting SAM files were transformed into BAM files using samtools. Duplicate reads were eliminated using Picard MarkDuplicates [20].

Whole genome sequencing (WGS) data includes mapping rate, genome coverage, mapping quality, and duplicate reads, as shown in Additional file 3 [15]. In the first experiment (Table 1, data set_1st analysis) [16, 18], Each sample produced approximately 6.2 million to a maximum of 7.7 million reads, an average mapping rate of more than 98%, and a mapping quality score of more than 31. Duplicate reads were about 13%, and the coverage was about 31 on average. In the second experiment, each sample produced approximately 7.2 million to a maximum of 8.4 million reads, an average mapping rate of more than 98%, and a mapping quality score of more than 31 (Table 1, data set_2nd analysis) [17, 18]. Duplicate reads were about 14%, and the coverage was about 35 on average. The sequencing coverage required to identify genomic variants may vary depending on the purpose. A coverage above 30X is sufficient to detect most variation, copy number variation (CNV), and structural variation in the genome [21].

## Limitations

Since the number of samples for each condition is small, performing a genome-wide association study to identify genomic regions associated with drug toxicity is challenging.

## Supplementary Information

**Additional file 1.** 1st analysis PLT counts.

**Additional file 2.** 2nd analysis PLT counts.

**Additional file 3.** Quality and quantity of the sequencing data.

## Data Availability

The Additional files 1, 2 and 3 described in this Data Note can be freely and openly accessed on FigShare (https://figshare.com/) [13–15]. Data set was deposited in the Korea Nucleotide Archive (KoNA, https://kobic.re.kr/kona) with open accession ID KRA2200718 and KRA2200719 [16, 17] and the NCBI Sequence Read Archive (SRA, https://www.ncbi.nlm.nih.gov/sra) with open accession ID SRP409432 [18].

## Abbreviations

ASO: Anti-Sense Oligonucleotide
WGS: Whole Genome Sequencing
GATK: Genome Analysis Tool Kit
